# Genome-Wide Identification and Characterization of KNOTTED-Like Homeobox (KNOX) Homologs in Garlic (*Allium sativum* L.) and Their Expression Profilings Responding to Exogenous Cytokinin and Gibberellin

**DOI:** 10.3390/ijms22179237

**Published:** 2021-08-26

**Authors:** Siyu Zhang, Yupeng Pan, Chengchen Zhi, Yujie Zheng, Xi’ao Wang, Xiaxia Li, Zhihui Cheng

**Affiliations:** College of Horticulture, Northwest A&F University, Yangling 712100, China; zhangsiyu@nwafu.edu.cn (S.Z.); yupeng.pan@nwafu.edu.cn (Y.P.); zhichengchen@nwafu.edu.cn (C.Z.); zyujie@nwafu.edu.cn (Y.Z.); wangxa@nwafu.edu.cn (X.W.); lixiaxia@nwafu.edu.cn (X.L.)

**Keywords:** garlic, *KNOX* genes, genome-wide identification, expression analysis

## Abstract

Garlic (*Allium sativum* L.) is an important vegetable and is cultivated and consumed worldwide for its economic and medicinal values. Garlic cloves, the major reproductive and edible organs, are derived from the axillary meristems. KNOTTED-like homeobox (KNOX) proteins, such as SHOOT MERISTEM-LESS (STM), play important roles in axillary meristem formation and development. However, the KNOX proteins in garlic are still poorly known. Here, 10 *AsKNOX* genes, scattered on 5 of the 8 chromosomes, were genome-wide identified and characterized based on the newly released garlic genome. The typical conserved domains of KNOX proteins were owned by all these 10 AsKNOX homologs, which were divided into two Classes (Class I and Class II) based on the phylogenetic analysis. Prediction and verification of the subcellular localizations revealed the diverse subcellular localization of these 10 AsKNOX proteins. Cis-element prediction, tissue expression analysis, and expression profilings in responding to exogenous GA_3_ and 6-BA showed the potential involvement of *AsKNOX* genes in the gibberellin and cytokinin signaling pathways. Overall, the results of this work provided a better understanding of *AsKNOX* genes in garlic and laid an important foundation for their further functional studies.

## 1. Introduction

Garlic, an economically important vegetable, spice, and medicinal crop, originated in central Asia and the Mediterranean and Caucasus zones [1]. Nowadays, as the second most important *Allium* crop after the bulb onion, garlic is cultivated and consumed worldwide for its remarkable medicinal and nutraceutical properties [2]. Commercial garlics are usually sterile and asexually reproduced with cloves or bulbils, which results in a relatively lower reproduction coefficient. Garlic clove, as the major reproductive and edible organ, contains a growth bud, a thick storage leaf, and a protective leaf, which is derived from the axillary meristem and similar to the tiller in rice or wheat and branch in woody plants [3]. Therefore, promoting the formation and development of axillary meristems in garlic is an effective way to increase clove numbers and further improve the reproduction coefficient. On the contrary, it is possible to obtain one-clove garlics by inhibiting the growth of garlic axillary meristems. Cytokinin (CK) has long been considered to be the key phytohormone to promote lateral bud outgrowth. Recently, researchers have reported that exogenous gibberellin (GA) was a positive regulator in lateral bud development [4], for example, GA_3_ treatment increased the number of tubers per plant in potato [5], induced tillering in welsh onions [6], and promoted shoot branching in *Jatropha carcass* [4]. Our previous work also indicated that exogenous GA_3_ with appropriate concentration, either injected into garlic plants or soaking the seed cloves before sowing, was able to significantly increase the cloves in a single garlic bulb [7,8]. Further histological and physiological analysis revealed that the exogenous GA_3_ promoted axillary bud formation and outgrowth via increasing the level of cytokinin (zeatin riboside, ZR) and soluble proteins and reducing the content of GA_3_, IAA, and sugar in the stems of the garlic plant, resulting in increased cloves per bulb [7,8].

Although the regulation of exogenous GA_3_ in promoting lateral bud formation and growth has been revealed from the aspects of histology and physiology, the related regulating or responding genes are still unknown in garlic. In plants, branching or tillering is achieved by axillary meristems, which are established in the axil of each leaf base. Research into axillary meristems revealed that several transcription factors (TFs) and phytohormones are key regulators that enable axillary meristem formation, in which the expression levels of *SHOOT MERISTEM-LESS* (*STM*) played essential roles [9]. STM is a member of the homeodomain (HD) KNOTTED-like homeobox transcription factors (KNOX) that regulate several processes of plant organ development such as meristem development, hormone metabolisms, and lignin biosynthesis [10]. *KNOX* genes belong to the group of TFs known as the three-amino acid loop extension (TALE) superclass [11] and usually contain 4 typical domains: KNOX1 domain, KNOX2 domain, ELK domain, and Homeobox KN domain [12], although some lack the ELK and Homeobox KN domains. The KNOX proteins, which have been thoroughly investigated, influence plant growth and development in a versatile context-dependent manner [13].

In Arabidopsis, there are eight KNOX homologs which have been divided into two Classes: Class I including KNAT1 (BP), KNAT2, KNAT6, and STM, and Class II containing KNAT3, KNAT4, KNAT5, and KNAT7 [14]. Studies on the functions of Class II KNOX proteins (KNAT3, KNAT4, KNAT5, and KNAT7) are less reported. The KNAT3, KNAT4, and KNAT5 might play distinct roles in root development [15], while the KNAT7 is involved in the fine regulation of secondary wall and lignin synthesis [16]. Additionally, a Class II KNOX protein, MdKNOX19, participating in the abscisic acid (ABA) pathway and affecting seed and fruit development, was recently characterized in apple [17]. For the Class I KNOX proteins, STM is essential for meristem formation and maintenance [13]; KNAT1 works redundantly with STM in the Arabidopsis shoot apical meristem (SAM) under the absence of *AS1*(*ASYMMETRIC LEAVES1*) [18] and takes part in controlling inflorescence stem development [19]; KNAT6, similar to KNAT1, contributes to meristem function and inflorescence development [20]; and, finally, KNAT2 regulates flower patterning acting in the inner whorls [21]. Furthermore, the Class I KNOX proteins were evidenced as the general orchestrators of growth-regulator homeostasis at the shoot apex by simultaneously activating cytokinin and repressing GA biosynthesis, thus promoting meristem activity in Arabidopsis [22]. Overall, KNOX proteins as versatile regulators play essential roles in sculpting plant form and its diversity [13].

Despite the identification, characterization, and functions of KNOX proteins that have been widely studied in Arabidopsis [12,22], poplar [23], peach [10], and pear [24], the KNOX homologs in garlic is unknown. Furthermore, whether the garlic AsKNOX proteins have the same function involved in the formation and outgrowth of axillary meristems and further regulate the cloves’ differentiation by interacting with phytohormones of gibberellin and cytokinin is also unclear. Genome-wide identification and characterization of AsKNOX proteins will provide us more information in understanding the potential functions of *AsKNOX* genes in regulating garlic axillary bud formation and outgrowth. Fortunately, the newly released chromosome-level garlic genome [2] provides a good opportunity to investigate the *KNOX* gene family members in garlic from a whole-genome perspective. Therefore, the objectives of the present study is to conduct a genome-wide characterization of *AsKNOX* gene families in the garlic genome and to reveal their expression profilings in responding to exogenous cytokinin and gibberellin. Results of this study not only provide target genes for the study of the *KNOX* family in garlic but also lay a foundation for the investigation of the molecular regulation of GA-induced garlic axillary meristem formation and further enrich our knowledge of improving garlic propagation efficiency via exogenous phytohormones application.

## 2. Results

### 2.1. Identification and Characterization of KNOX Family Members

Genome-wide identification of *KNOX* gene family members was performed with the newly assembled garlic genome [2], in which the HHM profiles of the conserved KNOX protein domains were firstly used as queries to perform an HMMER search (http://hmmer.org/, accessed on 15 January 2021). Then, the SMART search and Pfam search were performed to confirm the presence of the conserved domains for those potential garlic KNOX proteins. Finally, 10 *KNOX* genes were identified in the garlic genome. For convenience, these 10 genes were assigned names from *AsKNOX1* to *AsKNOX10* based on their chromosomal locations (Table 1). These *AsKNOX* genes encoded predicted peptides ranging from 160 to 354 aa with the isoelectric point (pI) value from 4.05 to 7.29 and the molecular weight (Mw) from 17.25 to 40.21 kDa. All AsKNOX homologs were predicted to be hydrophilic proteins and localized in the nucleus, of which one (AsKNOX1) and two (AsKNOX7 and AsKNOX10) members also showed localizations in chloroplast and cytoplasm, respectively (Table 1). All the related sequences information and detailed positions of these *AsKNOX* genes are listed in the Supplemental Table S1.

### 2.2. Chromosomal Distribution and Gene Structures of AsKNOX Genes

**Figure 1:**
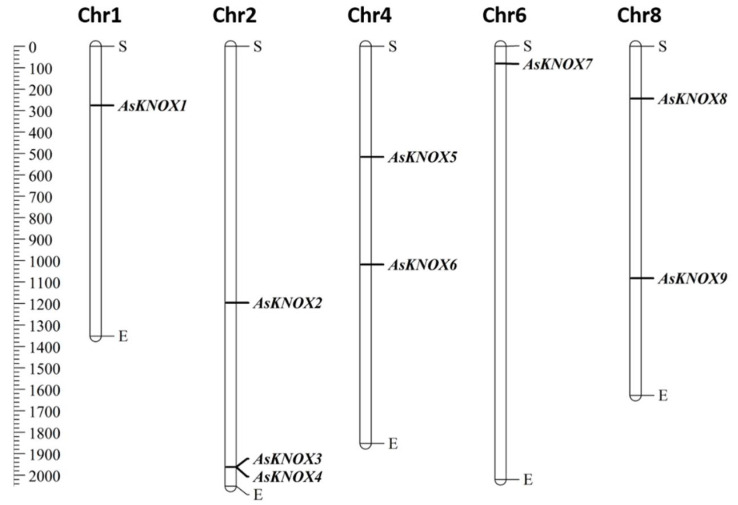
Distribution of *AsKNOX* genes on garlic chromosomes. The left scale represents the length of garlic chromosomes with a unit of Megabase pairs (Mb). The letters of S and E indicate the start and end positions of each chromosome.

Based on the physical locations in the garlic genome, nine of the ten *AsKNOX* genes (*AsKNOX1* to *AsKNOX9*) were anchored onto five of the eight chromosomes (Chr1, 2, 4, 6, and 8). However, *KNOX* gene *Asa0G05087.1* was not located on any assembled garlic chromosomes and was named with *AsKNOX10*. As shown in Figure 1, both Chr1 and Chr6 harbored one *AsKNOX* gene, while both Chr4 and Chr8 had two *AsKNOX* genes, and three *AsKNOX* genes were located on Chr2.

**Figure 2:**
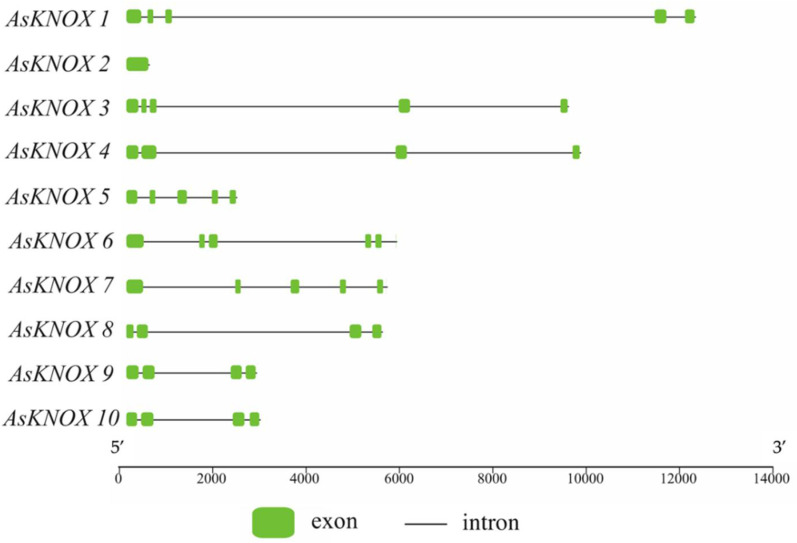
Exon-Intron structures of *AsKNOX* genes in garlic genome. The green boxes indicate the exons and the black lines indicate the introns. The bottom scale is represented with base pair (bp).

The Exon-Intron structures of *AsKNOX* genes were analyzed and plotted with TBtools. As shown in Figure 2, the exon numbers of these *AsKNOX* genes ranged from one to five, in which AsKNOX2 was the only member that had one exon. Five *AsKNOX* genes (*AsKNOX1*, *AsKNOX3*, *AsKNOX5*, *AsKNOX6*, and *AsKNOX7*) showed with the structures of 5 exons and 4 introns. In addition, all the other four *AsKNOX* genes (*AsKNOX4*, *AsKNOX8*, *AsKONX9*, and *AsKNOX10*) had four exons and three introns. Among these *AsKNOX* genes, *AsKNOX9* and *AsKNOX10* had similar gene structures with each other, which might suggest that these two genes have similar functions in garlic. Additionally, the gene structure of *AsKNOX3* was also somewhat similar to that of *AsKNOX4*, although the 2nd exon of *AsKNOX4* was spliced into two small exons (2nd and 3rd exon) in *AsKNOX3*.

### 2.3. Cis-Element and Conserved Motif Analysis of AsKNOX Genes

**Figure 3:**
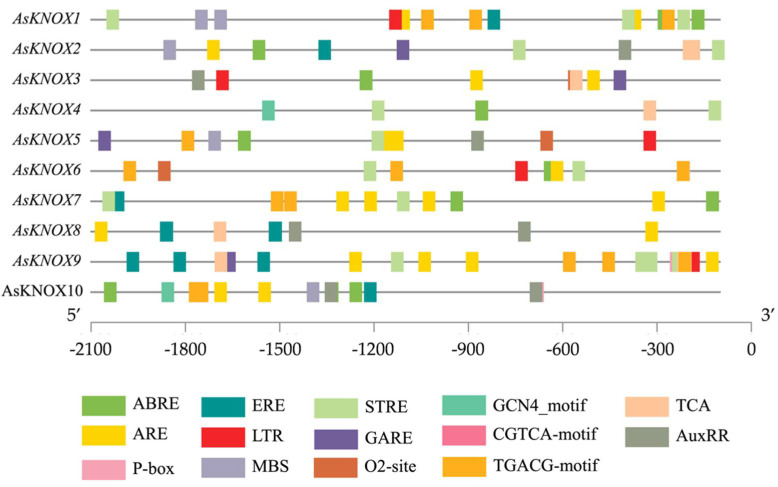
Putative cis-elements in the promoter regions of *AsKNOX* genes. Different colored rectangles denote different cis-elements with various biological functions.

To further explore the possible regulation mechanism of *AsKNOX* genes in garlic, the cis-elements in their promoter regions were scanned in the database of PlantCARE. In total, 14 cis-elements were obtained and listed in Figure 3, which included two development-related, four stress responses-related, and seven phytohormone-related cis-elements. The development-related cis-elements (O2-site and GCN4-motif) are relevant to zein metabolism regulation and endosperm expression, respectively. The stress response-related cis-elements included the MYB binding site involved in drought-inducibility (MBS), anaerobic induction element (ARE), low-temperature responsiveness (LTR), and stress response element (STRE). The phytohormone-related cis-elements are involved in several hormone signalings, such as the abscisic acid (ABRE), auxin (AuxRR), gibberellin (GARE and P-box), jasmonic acid (CGTCA-motif and TGACG-motif), and ethylene (ERE). Moreover, the TCA cis-element was also presented in the promoters of *AsKNOX* genes. These findings might indicate the involvement of *AsKNOX* genes in the regulation of development and responses to abiotic stresses and phytohormones.

**Figure 4:**
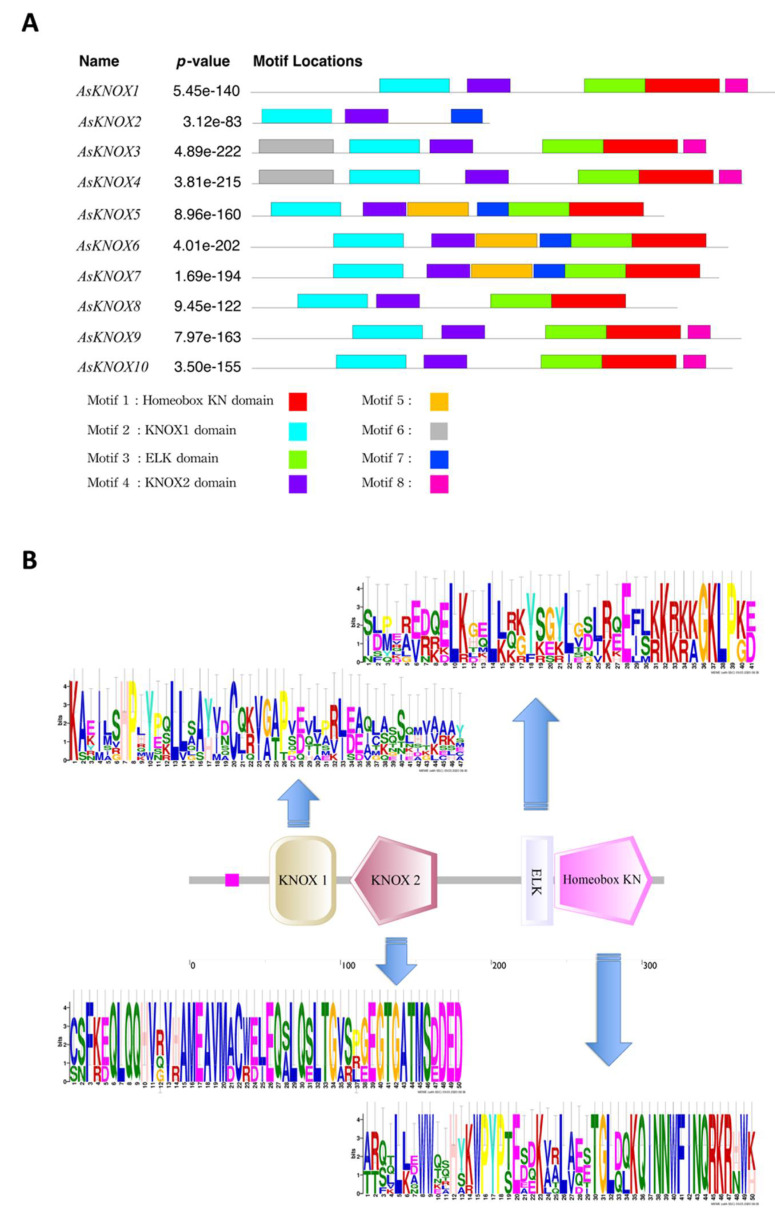
Conserved motif analysis of garlic AsKNOX homologs. (**A**) The conserved motifs within the 10 garlic AsKNOX proteins. Motif 1 to 8 were indicated with different colors; (**B**) The stacked motif logos of the KNOX1 domain, KNOX2 domain, ELK domain, and Homeobox KN domain.

Furthermore, to obtain insights into the diversity of conserved motif compositions in AsKNOX proteins, the protein sequences of these 10 *AsKNOX* genes were assessed using the MEME program. As shown in Figure 4, eight conserved motifs were identified among these 10 AsKNOX proteins, of which the first four motifs (motif 1 to 4) correspond to the 4 typical KNOX domains (Homeobox KN domain, KNOX1 domain, ELK domain, and KNOX2 domain). These 4 typical domains of KNOX protein were owned by all the AsKNOX proteins, except for AsKNOX2, which contains 2 of these 4 typical domains (KNOX1 domain and KNOX2 domain). Additionally, the remaining four motifs (motif 5 to 8) were scattered among nine of the 10 AsKNOX homologs (Figure 4A). Furthermore, based on the stacked motif logos of those four typical domains among AsKNOX proteins, the KNOX2 domain, and Homeobox KN domain showed relatively more conservation than the KNOX1 domain and ELK domain (Figure 4B) among these 10 AsKNOX proteins. To more accurately understand the specific positions of these conserved domains in their protein sequences, the Pfam website was used to compare the sequences and to retrieve the detailed positions of each domain, which are listed in Supplemental Table S1.

### 2.4. Phylogenetic Analysis of AsKNOX Homologs

**Figure 5:**
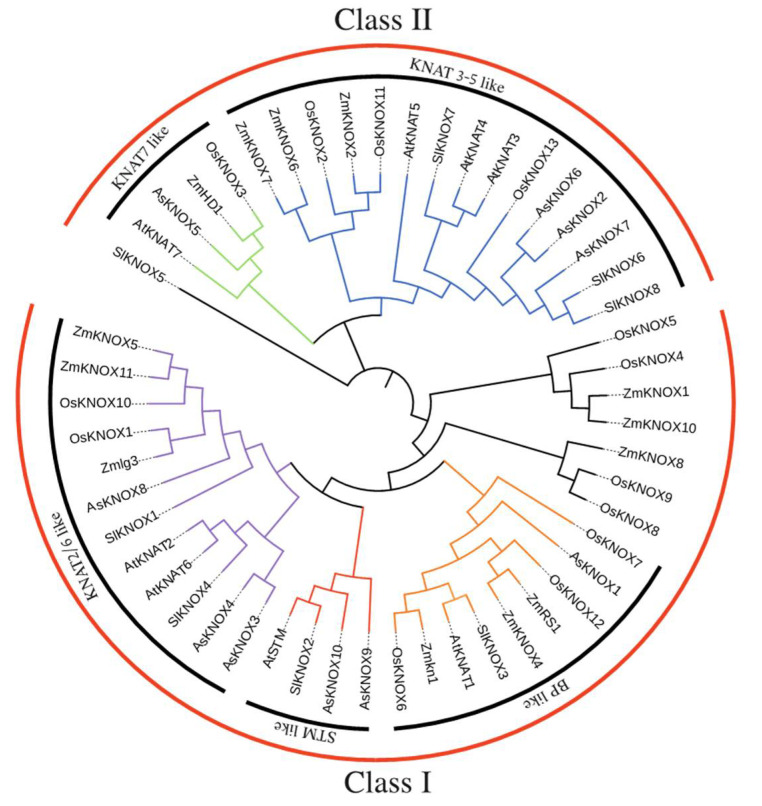
Interspecific phylogenetic tree of KNOX proteins from various species. A phylogenetic tree was constructed using the neighbor-joining method with bootstrap test (1000 iterations) by MEGA X.

To clarify the evolutionary relationships of 10 AsKNOX proteins and to predict their potential biological functions, a phylogenetic tree was constructed based on the alignment of protein sequences including the KNOX proteins collected from Arabidopsis, tomato, rice, and maize (Figure 5 and Table S2). The neighbor-joining (NJ) phylogenetic tree divided the KNOX proteins into two groups, Class I and Class II, in which Class I includes six AsKNOX members (AsKNOX1, AsKNOX3, AsKNOX4, AsKNOX8, AsKNOX9, and AsKNOX10), while the remaining four AsKNOX homologs (AsKNOX2, AsKNOX5, AsKNOX6, and AsKNOX7) belong to the Class II. According to the classification method reported by Cheng et al. [24], these proteins were further divided into 5 subgroups: Class I includes STM-like, BP-like, and KNAT2/6-like subfamilies; Class II consists of KNAT7-like and KNAT3-5-like subfamilies. Among these 10 AsKNOX proteins, both AsKNOX9 and AsKNOX10 belong to the STM-like subfamily; AsKNOX1 is a member of the BP-like subfamily; AsKNOX3, AsKNOX4, and AsKNOX8 belong to the KNAT2/6-like subfamily; AsKNOX2, AsKNOX6, and AsKNOX7 are classified into the KNAT3-5-like subfamily, while AsKNOX5 belongs to KNAT7-like subfamily.

### 2.5. Expression Patterns of AsKNOX Genes in Different Garlic Tissues

**Figure 6:**
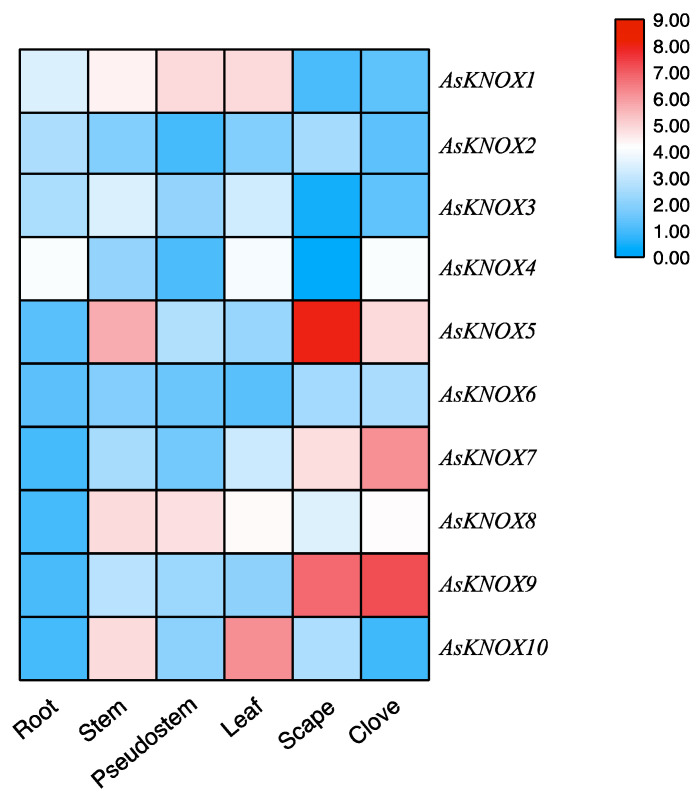
Expression analysis of *AsKNOX* genes in different garlic tissues. The color scale from blue to red represents the lower to higher relative expression levels.

To primarily investigate the functions of *AsKNOX* genes, their expression patterns were analyzed among six different garlic tissues including root, stem, pseudostem, leaf, scape, and clove. As shown in Figure 6, different expression patterns were found for these 10 *AsKNOX* genes. *AsKNOX1*, *AsKNOX5*, *AsKNOX8* and *AsKNOX10* were expressed in stem that contains the apical meristem of garlic. Both *AsKNOX1* and *AsKNOX8* were expressed in the pseudostem and leaf, while *AsKNOX8* was barely expressed in root and *AsKNOX1* had rarely expression in both scape and clove. *AsKNOX2* showed low expression levels among all the investigated garlic tissues, especially for the pseudostem and clove. Compared with other garlic tissues, *AsKNOX6* had more rarely expressions in root and leaf. Expressions of *AsKNOX4* in the root, leaf, and clove were higher than other tissues, especially for the scape. Similarly, *AsKNOX3* also showed very low expression in the scape. *AsKNOX5*, *AsKNOX7*, and *AsKNOX9* were all highly expressed in garlic scape and clove, and higher expression in garlic stem was also found for *AsKNOX5*. The expression levels of *AsKNOX10* were much higher in garlic stem and leaf, while almost no expressions of *AsKNOX10* were shown in garlic root and clove.

### 2.6. Responses of AsKNOX Genes to Exogenous GA_3_ and 6-BA Treatments

**Figure 7:**
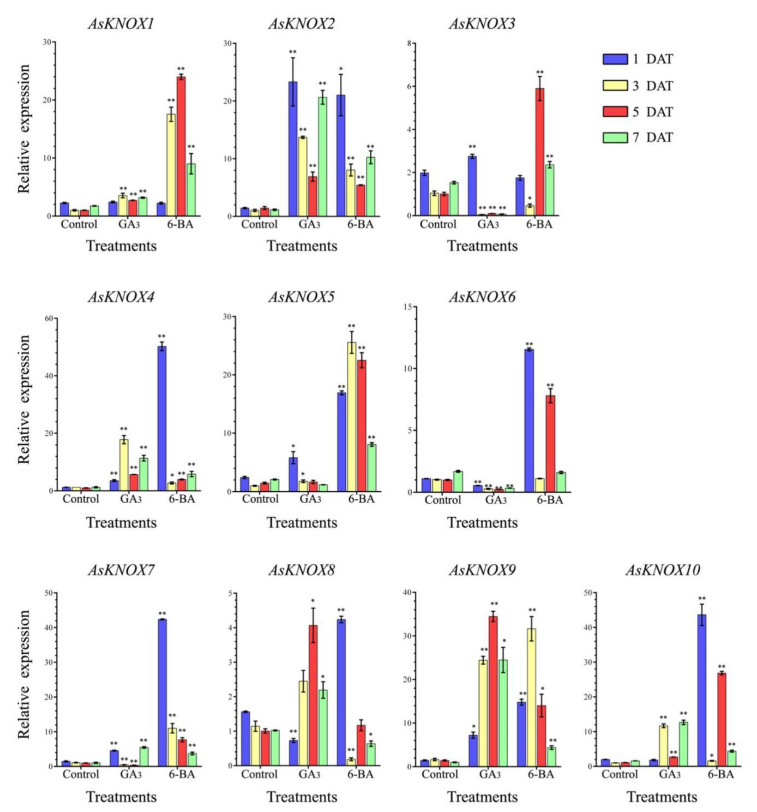
Expression analysis of the *AsKNOX* genes under exogenous GA_3_ and 6-BA treatments. Student’s t-test was used to determine significant differences at the same period between control group and treatment group. Control means the control without any treatment, GA_3_ means treated with exogenous GA_3_ solution, 6-BA means treated with exogenous 6-BA solution. Significance level: * *p* < 0.05. ** *p* < 0.01.

To reveal the potential responses of *AsKNOX* genes to exogenous GA_3_ and 6-BA, the expression patterns of these 10 *AsKNOX* genes were checked in the garlic stems that were collected at the 1, 3, 5, and 7 DAT (Days after treatment) (Figure 7). These 10 *AsKNOX* genes had different expression patterns under either GA_3_ or 6-BA treatments.

When treated with GA_3_, the expressions of *AsKNOX1*, *AsKNOX2*, *AsKNOX4*, and *AsKNOX9* showed more significant increases than those of their controls. Compared with the control groups, the expression of *AsKNOX3* was up-regulated at the 1 DAT, while it was down-regulated at the rest sample points. The expression of *AsKNOX5* was more significantly increased than that of the control at both 1 DAT and 3 DAT. The *AsKNOX6* had different expressions which were significantly decreased at all sample points compared with the control groups. *AsKNOX7* showed obvious increases at the 1 DAT and 7 DAT, while decreased expressions were obtained at 3 DAT and 5 DAT. Compared with their controls, the expressing of *AsKNOX8* was significantly down-regulated at the 1 DAT, while *AsKNOX10* had no obvious changes at this sample point. Both the expressions of *AsKNOX8* and *AsKNOX10* were up-regulated compared with their controls at all other sample points (3, 5, and 7 DAT).

For the 6-BA treatments, the expressions of *AsKNOX2, AsKNOX4, AsKNOX5, AsKNOX7, AsKNOX9*, and *AsKNOX10* showed obvious increases than their controls at all sample points. *AsKNOX1* and *AsKNOX3* had no obvious changes compared with their controls at the 1 DAT, while the expressions of *AsKNOX1* and *AsKNOX3* at the 3 DAT were significantly increased and decreased than their controls, respectively. Expressions of *AsKNOX6* had no obvious difference compared with its controls for the 3 DAT and 7 DAT samples, while its expressions were significantly increased compared with the controls at 1 DAT and 5 DAT. For the 3 DAT and 7 DAT samples, the expressions of *AsKNOX8* were obviously decreased compared with its controls.

### 2.7. Subcellular Localization of AsKNOX8 and AsKNOX6 Proteins

**Figure 8:**
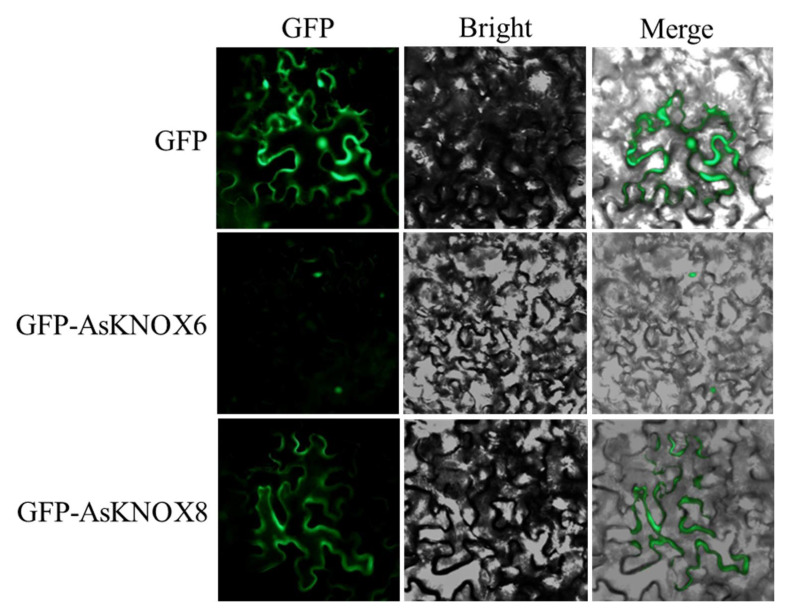
Subcellular localizations of AsKNOX8 and AsKNOX6 proteins. Both the GFP protein and the fusion proteins GFP-AsKNOX8 and GFP-AsKNOX6 were transiently expressed in the epidermal cells of tobacco leaves. Green fluorescence, bright-field, and merged images were orderly shown from left to right.

To preliminarily verify the results of predicted subcellular localization, two family members were randomly selected for verification. The subcellular localization of AsKNOX6 and AsKNOX8 proteins were verified with transient expression of GFP fusion proteins GFP-AsKNOX6 and GFP-AsKNOX8 in tobacco leaf cells. The results of the control experiment showed that the fluorescence signal of GFP could be detected in both the nucleus and cytoplasm of tobacco cells (Figure 8).

The GFP signal of GFP-AsKNOX6 was successfully observed in the nucleus, which was consistent with the predicted results in Table 1. However, the GFP signal of GFP-AsKNOX8 was unexpectedly localized on the cytoplasm.

## 3. Discussion

The first reported *KNOX* gene in plants was the *ZmKn1* from maize [25], which is involved in the maintenance of the shoot apical meristem and is related with the cell fates switching from indeterminate to determinate [26]. Subsequently, more *KNOX* genes were identified and characterized in other plant species [13]. The *KNOX* genes seem to have a limited number in different species, for example, 8 in Arabidopsis, 13 in rice, 12 in maize, 15 in poplar [12], 10 in peach [10], 22 in apple [27], and 18 in pear [24]. Furthermore, the number of *KNOX* genes among different plant species seems not to correlate with their genome sizes, which was further confirmed in the present study. Although garlic has a genome size of ~16 GB, here only 10 *AsKNOX* genes were genome-wide identified. These *AsKNOX* genes are scattered on 5 garlic chromosomes (Chr1, 2, 4, 6, and 8) and subcellular localization prediction of these 10 AsKNOX proteins suggested that all of them could have nuclear localizations. In addition, the results also suggested that AsKNOX1, AsKNOX7, and AsKNOX10 could have locations on the chloroplast or cytoplasm. Intriguingly, verification of subcellular locations of AsKNOX6 and AsKNOX8 showed that AsKNOX6 was consistently localized in the nucleus, while AsKNOX8 was unexpectedly localized on the cytoplasm (Figure 8). The subcellular localization of KNOX proteins outside the nucleus was also found in other plant species, such as the MdKNOX15 in apple located in both the nucleus and plasma membrane [28] and the KNAT1 in Arabidopsis when co-expressed with AtOFP1 or AtOFP5, in which the AtOFP negatively controls the activity of KNOX-BELL protein dimers by changing their localization from the nucleus to the cytoplasmic space [29]. Transcription factors might be retained outside the nucleus for several potential reasons, for examples containing membrane-binding domains, interacting with protein partners that inhibit nuclear import or directly interplaying with membranes, and the posttranslational modifications (phosphorylation, acetylation, glycosylation, ubiquitination, sumoylation, and redox-dependent changes) [30]. Therefore, the cytoplasm localization of AsKNOX8 might indicate that it works with other proteins to play its regulatory function. Another possible reason for the subcellular localization of AsKNOX proteins located outside the nucleus probably was the intercellular transport of KNOX transcription factors. For example, the microinjection studies performed by Lucas et al. (1995) showed that encoded knotted1 (kn1) of maize can be transported to adjacent cells by plasmodesmata [31].

Four typical conserved domains including Homeobox KN domain, KNOX1 domain, ELK domain, and KNOX2 domain were usually contained in KNOX proteins [12]. Similarly, all the AsKNOX homologs in garlic also showed these four typical domains, except for the AsKNOX2 which has not the Homeobox KN domain and ELK domain. Multiple sequence alignment and phylogenetic analyses of these AsKNOX proteins revealed that these 10 AsKNOX, similar to Arabidopsis, can be divided into two classes (Class I and II) and five subfamilies (STM-like, BP-like, KNAT2/6-like, KNAT3-5-like, and KNAT7-like). Among these 10 AsKNOX proteins, both AsKNOX9 and AsKNOX10 belong to the STM-like subfamily; AsKNOX1 is a member of the BP-like subfamily; AsKNOX3, AsKNOX4, and AsKNOX8 belong to the KNAT2/6-like subfamily; AsKNOX2, AsKNOX6, and AsKNOX7 are classified into the KNAT3-5-like subfamily, while AsKNOX5 belongs to KNAT7-like subfamily. In Arabidopsis, the Class I *KNOX* genes (*STM*, *BP*/*KNAT1*, *KNAT2*, and *KNAT6*) play important roles in the maintenance of meristematic potentials and are expressed mainly in the meristematic regions, less in differentiated tissues, and not at all in the mature organs [32]. However, the Class II *KNOX* genes (*KNAT3*, *KNAT4*, *KNAT5*, and *KNAT7*) of Arabidopsis, showing functions in root [15] and lateral organ differentiation [14] and secondary cell wall biosynthesis [33], have expressions in both differentiating tissues and mature organs, but not in the meristematic zones [14]. Tissues expressions of these 10 *AsKNOX* genes indicated that the expressions of *AsKONX* genes might not strictly follow the tissue expression patterns of Arabidopsis, in which either the members of Class I and II *AsKNOX* genes, for example, *AsKNOX5* and *AsKNOX9*, showed expressions in mature organs such as the garlic scapes and cloves. *AsKNOX1*, *AsKNOX8*, and *AsKNOX10* showed relatively higher expressions in the organs derived from the apical meristem tissues such as garlic stems and leaves. Since the stem of garlic is a condensed stem, both the apical and axillary meristems are growing in this part. Therefore, the *AsKNOX* genes with higher expressions in garlic stems (*AsKNOX1*, *8*, and *10*), perhaps have similar functions with the Arabidopsis Class I *KNOX* genes that play roles in the meristem maintenance. The high expressions of *AsKNOX5* and *AsKNOX9* in garlic scape might suggest their functions in the development of garlic inflorescences, which might be similar to the *KNAT7* and *STM* in Arabidopsis, that repress the cell wall biosynthesis of inflorescence stems and influence the development of inflorescence architecture, respectively [34,35].

Research work has indicated that *KNOX* genes regulate the metabolic activities and signaling pathways associated with several phytohormones [36], including cytokinin, gibberellin, auxin, brassinosteroid, and abscisic acid. In Arabidopsis, KNOX function is mediated by cytokinin (CK) which is a growth regulator that promotes cell division and meristem function [22]. KNOX proteins regulate the balance between gibberellin (GA) and CK in the shoot apex to prevent cell differentiation and thus maintain pluripotent cell fate in the shoot apex meristem [37]. Furthermore, both in the natural context of KNOX function in the SAM and upon ectopic KNOX expression in Arabidopsis leaves, reducing activity of the GA growth regulators could promotes meristematic activity [38]. Recently, studies in apple indicated that expressions of *KNOX* genes are also inducible by exogenous phytohormones [17,27]. For example, *MdKNOX19*, a Class II *KNOX* gene regulating apple fruit and seed developments through ABA signaling, was significantly up-regulated when applied ABA to apple leaves, fruits, and seeds [17]. Furthermore, expressions of the fourteen *MdKNOX* genes that strongly exhibited expressions in the floral bud of apple were also more or less affected by the applications of exogenous phytohormones of 6-BA, GA_3_, ABA, and SA during the flower induction period [27]. In the present study, phytohormone-associated cis-elements, including auxin, abscisic acid, gibberellin, and ethylene-responsive elements were also shown within the promoters of *AsKNOX* genes (depicted in Figure 3), which might indicate the involvement of *AsKNOX* genes in these related phytohormone signaling pathways. Additionally, several abiotic stress responsive elements were also predicted in the promoters of *AsKNOX* genes, which suggest that *AsKNOX* genes might also contribute to the resistance of garlic to abiotic stresses.

In Arabidopsis, the KNOX Class I transcription factors have functions in repressing GA synthesis and promoting cytokinin synthesis, respectively, to maintain the meristem identity [13]. Furthermore, lateral organ founder cells with low CK and high GA could lead to the absence of *KNOX* expression [22,37]. To reveal the potential function of *AsKNOX* genes responsive to GA and CK, we checked the expression profilings of the identified 10 *AsKNOX* genes under either exogenous GA_3_ or 6-BA treatments. Compared with the untreated control groups, expressions of *AsKNOX1*, *AsKNOX5,* and *AsKNOX7* showed a significant increase under two treatments, while these *AsKNOX* genes showed much higher expressions in their 6-BA treatments than their GA_3_ treatments. The expression levels of *AsKNOX3*, *AsKNOX6,* and *AsKNOX7* under these two treatments showed opposite trends, for example the expression of *AsKNOX6* decreased at all sample points when treated with GA_3_ but significantly increased under the 6-BA treatment. Conversely, *AsKNOX2*, *AsKNOX4*, and *AsKNOX9* showed identical expression patterns in response to 6-BA and GA_3_ treatments, and the transcription of these *AsKNOXs* were strongly induced by both 6-BA and GA_3_. These results were similar to a previously reported study, for example, the transcription of *MdKNOX1/20* was strongly induced by both exogenous 6-BA and exogenous GA_3_ at 30 and 70 days after full bloom in apple [27]. For both the treatments of exogenous GA_3_ and 6-BA, *AsKNOX* genes showed relatively complicated expression patterns at the sampling points of 1, 3, 5, and 7 DAT. This might indicate that each of the *AsKNOX* genes perhaps has a different regulating function in garlic. Notably, the relative expression levels of *AsKNOX9* and *AsKNOX10* under the GA_3_ treatment were significantly higher than their control groups. This might differ from previous studies of the *KNOX* gene family in other plants such as the Arabidopsis. Despite this, more research still needs to be conducted to reveal the detailed functions of *AsKNOXs* in garlic either from the perspective of the organ development (such as the differentiation of garlic cloves) or from the side of abiotic stresses responsiveness.

## 4. Materials and Methods

### 4.1. Plant Materials and Phytohormone Treatments

The garlic cultivar G024, widely cultivated in the northwest of China, was used in this study. Bulbs of G024 show the following morphological characteristics: fresh weight 30 to 50 g, bulb diameter 3.7 to 5.7 cm, and 10 to 12 cloves arranged in two whorls for each bulb. The seed cloves of G024 were provided by the Vegetable Physiology and Biotechnology Laboratory of Northwest A&F University. At the beginning of September 2019, the healthy and uniform-sized cloves of G024 were selected and planted in the Wuquan Experiment Station of Northwest A&F University in Yangling, China. Throughout the growing period, standard agronomic practices were performed to maintain the garlic plants.

To evaluate the expression patterns of the *AsKNOX* genes, their expression levels were checked in six kinds of organs or tissues of garlic. The samples of garlic leaf, stem, pseudostem, and root were collected on the 90th day after planting. The stems containing meristems were sampled with a height of 1 cm above the root. The pseudostem samples were cut from the lower part of the leaf, also with a height of 1 cm. The scape samples were collected on the 215th day after planting and the clove samples were collected from the seed cloves. All the samples were separated from garlic plants using scalpels and tweezers. To reveal the potential responses of *AsKNOX* genes to gibberellin and cytokinin, the exogenous phytohormones GA_3_ (1 mmol/L) and 6-BA (1 mmol/L) were used to treat garlic plants on the 90th day after planting. The treatment method strictly followed Liu et al. (2019) [7], in which the prepared phytohormones were injected into the stems from the top of garlic plants. Since the research of Liu et al. (2019) [7] also showed no difference in bulb structure between non-injection and injection of water treatments, so the control group of this study was directly performed with non-injection. Garlic stem samples were collected at the 1, 3, 5, and 7 days after treatment (DAT) to check the expression levels of all *AsKNOX* genes. The stem samples with a height of 1 cm and containing meristems were separated from garlic plants through the same method as before. All the collected tissue samples were immediately frozen with liquid nitrogen and then stored in a −80 °C freezer for further use.

### 4.2. Identification and Sequence Analysis of AsKNOX Family Members

The newly released garlic genome was used for *AsKNOX* genes genome-wide identification and it is available at the following website: https://doi.org/10.6084/m9.figshare.12570947.v1 (accessed on 5 November 2020). The typical conserved domain sequences of KNOX in Arabidopsis were downloaded from the website of TAIR (http://www.arabidopsis.org/, accessed on 1 January 2021). In addition, the HMM file built based on KNOX1 (PF03790) domain was used as a query to search the garlic local protein database by HMMER3.1 (E-value = 0.01). Other typical domains of KNOX proteins including KNOX2 (PF03791), ELK(PF03789), and Homeobox KN (PF05920) were further checked for the identified candidate homologs using the SMART (http://smart.embl-heidelberg.de/, accessed on 15 January 2021) and the Pfam (http://pfam.xfam.org/, accessed on 15 January 2021) databases. The position of each domain of AsKNOX proteins was also predicted with Pfam. Only those proteins containing at least two of these four conserved domains were considered as members of garlic AsKNOX. After removing the repeated and redundant sequences, the isoelectric point (pI) and molecular weight (MW) of each AsKNOX protein were predicted by using the ExPASy Proteomics Server (https://www.expasy.org/, accessed on 30 January 2021). Additionally, the subcellular localization of these AsKNOX proteins were predicted using the WoLF PSORT (https://www.genscript.com/wolf-psort.html/, accessed on 30 January 2021). All the related obtained information of these *AsKNOX* genes are listed in the Supplemental Table S1.

### 4.3. Chromosome Distribution and Gene Structure Analysis

According to the physical locations of each gene on the draft garlic genome (https://doi.org/10.6084/m9.figshare.12570947.v1, accessed on 5 November 2020), the identified *AsKNOX* genes were mapped onto the corresponding garlic chromosomes using the Mapchart (https://www.wur.nl/en/show/Mapchart.htm, accessed on 5 February 2021). The detailed positions of these genes are also provided in Table S1. Gene structures of these *AsKNOX* genes were analyzed using the TBtools software (https://github.com/CJ-Chen/TBtools/%20releases, accessed on 6 February 2021). Conserved motif structures were identified using Multiple EM for Motif Elicitation (MEME, http://meme-suite.org/, accessed on 7 February 2021), in which the parameter of a motif width larger than four and less than 100 was used. The potential cis-regulatory elements were analyzed by scanning the 2.0 kb upstream sequences from the transcription start codon ATG with the PlantCARE website (http://bioinformatics.psb.ugent.be/webtools/plantcare/html/, accessed on 10 February 2021).

### 4.4. Phylogenetic Analysis and Multiple Sequence Alignment

Based on the previously reported literature, we collected the KNOX proteins of different species [12,39,40] including rice, maize, tomato, and Arabidopsis. The sequences of these KNOX proteins were downloaded from the NCBI database (https://www.ncbi.nlm.nih.gov/, accessed on 15 February 2021), in which the detailed sequence accession numbers were listed in Table S2. Multiple sequence alignments of all these obtained KNOX proteins were performed by CLUSTALW (https://www.genome.jp/tools-bin/clustalw/, accessed on 15 February 2021). A phylogenetic tree was constructed using MEGA X, in which the neighbor-joining (NJ) method was used with a 1000-iterations bootstrap test.

### 4.5. RNA Isolation and Gene Expression Analysis

Total RNA was isolated from different garlic tissues using a Plant RNA Kit (Omega Bio-Tek) following the manufacturer’s instructions. Subsequently, the first-strand cDNA synthesis was performed using the Easy Script One-Step gDNA Removal and cDNA Synthesis Super Mix Kit (Trans Gen Biotech Co., Ltd., Beijing, China). The primers used for quantitative real-time PCR (qPCR) analysis were designed with Primer 3 Input (http://bioinfo.ut.ee/primer3-0.4.0/, accessed on 5 April 2021). Sequence information of all these primers were provided in Table S3. qPCR was conducted using the 2 × Sybr Green qPCR mix (Aidlab Biotechnologies Co., Ltd., China) in the CFX96 Touch Real-Time PCR Detection System (Bio-Rad) with the following procedures: 95 °C for 3 min; 95 °C for 15 s, 60 °C for the 30 s with 40 cycles. Relative quantification was calculated according to the 2^−ΔΔCT^ method described by Livak and Schmittgen [41], in which the garlic *ACTIN* gene (*AsACT*) was used as the reference gene [42]. Each PCR assay was run with three independent biological and technological replicates. The expression levels of each *KNOX* gene among different garlic tissues were displayed with a heatmap using the TBtools software (https://github.com/CJ-Chen/TBtools, accessed on 25 April 2021). The significance tests between the control and the exogenous phytohormones treatments were performed with the *t*-test analysis.

### 4.6. Verification of Subcellular Localization

**Figure 9:**
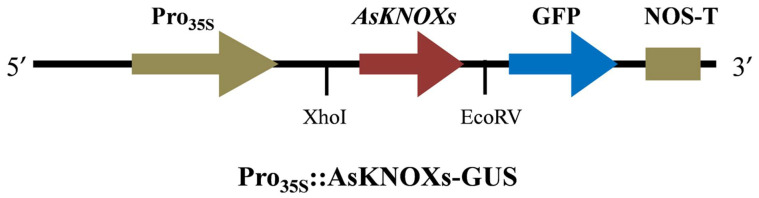
The connection mode of *AsKNOX* genes and vector.

To preliminarily check the predicted subcellular localizations of AsKNOX proteins, two of them (AsKNOX6 and AsKNOX8) were randomly selected for experimental verification. Full-length coding sequences of *AsKNOX* genes without stop codons were amplified using gene-specific primers and they were ligated into pGreen 0229 vector containing the green fluorescent protein (GFP) to generate GFP-AsKNOX fusion proteins. Sequence information of all these primers were provided in Table S4. The connection mode of *AsKNOX* genes and vector was shown in Figure 9. The Gene Star Seamless Cloning Kit was used to fuse the *AsKNOX* gene sequences to the linearized plasmid that was obtained through the double enzyme digestion with Xhol and EcoRV. The recombinant constructs were checked by sanger sequencing and were transfected into the abaxial side of 4- to 6-week-old *N. benthamiana* leaves by infiltration with strain GV3101 (*Agrobacteriumt tumefaciens*). The GFP empty vector was served as the positive control. After 48 h co-infiltration, the GFP signals in *N. benthamiana* leaves were observed using an automatic fluorescence microscope (Olympus, Tokyo, Japan) with 488-nm excitation.

## 5. Conclusions

Ten *AsKNOX* genes were genome-wide identified and characterized in the garlic genome. Their gene structure, cis-elements, conserved domains, subcellular localizations, phylogenetic relationships, chromosome locations, and expression profiles in different tissues and in responding to exogenous GA_3_ and 6-BA treatments were systematically analyzed in the present study. These 10 *AsKNOX* genes showed different expression patterns among garlic tissues and most of them had responsiveness under the treatments of exogenous GA_3_ and 6-BA, which might indicate that the involvement of *AsKNOX* genes in the gibberellin and cytokinin signaling pathways. Overall, our work laid a foundation for the further functional study of *AsKNOX* genes in garlic, which might also enlighten the potential roles of *AsKNOX* genes in regulating the GA_3_-promoted garlic clove differentiations and developments.

## Figures and Tables

**Table 1:** Information of 10 *AsKNOX* gene homologs in garlic genome.

Gene	Gene ID	Protein Length (aa)	pI	Mw (kDa)	GRAVY	Predicted Subcellular Localization
*AsKNOX1*	*Asa1G01043.1*	354	7.29	40.21	−0.961	Nucleus, Chloroplast
*AsKNOX2*	*Asa2G04444.1*	160	4.05	17.25	−0.367	Nucleus
*AsKNOX3*	*Asa2G07204.1*	307	5.10	34.65	−0.815	Nucleus
*AsKNOX4*	*Asa2G07205.1*	331	5.33	37.68	−0.684	Nucleus
*AsKNOX5*	*Asa4G01931.1*	278	5.86	32.13	−0.674	Nucleus
*AsKNOX6*	*Asa4G03695.1*	322	5.61	36.62	−0.752	Nucleus
*AsKNOX7*	*Asa6G00294.1*	315	5.68	35.84	−0.793	Nucleus, Cytoplasm
*AsKNOX8*	*Asa8G00804.1*	287	5.08	32.42	−0.592	Nucleus
*AsKNOX9*	*Asa8G04023.1*	330	6.08	37.41	−0.604	Nucleus
*AsKNOX10*	*Asa0G05087.1*	324	6.39	37.27	−0.785	Nucleus, Cytoplasm

## Data Availability

Not applicable.

## Supplementary Materials

The following are available online at https://www.mdpi.com/article/10.3390/ijms22179237/s1, Table S1: Informations of 10 *AsKNOX* genes. Table S2: Information of KNOX proteins used for phenlogenitc tree construction. Table S3: Primers used for qPCR analyis in this study. Table S4: Sequencesns of primers used for gene cloning and vector construction.

## References

[1] Etoh T., Watanabe H., Iwai S. (2001). RAPD variation of garlic clones in the center of origin and the westernmost area of distribution. Mem. Fac. Agric..

[2] Sun X., Zhu S., Li N., Cheng Y., Liu T. (2020). A chromosome-level genome assembly of garlic (*Allium sativum* L.) provides insights into genome evolution and allicin biosynthesis. Mol. Plant.

[3] Kamenetsky R. (2007). Garlic: Botany and Horticulture.

[4] Ni J., Gao C., Chen M.S., Pan B.Z., Ye K., Xu Z.F. (2015). Gibberellin promotes shoot branching in the perennial woody plant *Jatropha curcas*. Plant Cell Physiol..

[5] Alexopoulos A.A., Akoumianakis K.A., Passam H.C. (2010). The effect of the time and mode of application of gibberellic acid on the growth and yield of potato plants derived from true potato seed. Russ. J. Appl. Chem..

[6] Yamazaki H., Shiraiwa N., Itai A., Honda I. (2015). Involvement of gibberellins in the regulation of tillering in welsh onion (*Allium fistulosum* L.). Hortic. J..

[7] Liu H., Deng R., Huang C., Cheng Z., Meng H. (2019). Exogenous gibberellins alter morphology and nutritional traits of garlic (*Allium sativum* L.) bulb. Sci. Hortic..

[8] Liu H.J., Huang C.P., Tong P.J., Yang X., Cheng Z.H. (2020). Response of axillary bud development in garlic (*Allium sativum* L.) to seed cloves soaked in gibberellic acid (GA_3_) solution. J. Integr. Agric..

[9] Wang Y., Jiao Y. (2018). Axillary meristem initiation-a way to branch out. Curr. Opin. Plant Biol..

[10] Giulio T., Emiliano C., Ignazio V., Chiara N., Emilia C., Teresa D.M., Elisa V., Leonardo B., Beatrice B.M., Giovanni M. (2012). The peach (*Prunus persica* L. Batsch) genome harbours 10 *KNOX* genes, which are differentially expressed in stem development, and the class 1 KNOPE1 regulates elongation and lignification during primary growth. J. Exp. Bot..

[11] Bürglin T. (1997). Analysis of TALE superclass homeobox genes (MEIS, PBC, KNOX, Iroquois, TGIF) reveals a novel domain conserved between plants and animals. Nucleic Acids Res..

[12] Mukherjee K., Brocchieri L., Bürglin T.R. (2009). A comprehensive classification and evolutionary analysis of plant homeobox genes. Mol. Biol. Evol..

[13] Hay A., Tsiantis M. (2010). *KNOX* genes: Versatile regulators of plant development and diversity. Development.

[14] Furumizu C., Alvarez J.P., Sakakibara K., Bowman J.L., Qu L.J. (2015). Antagonistic roles for *KNOX1* and *KNOX2* genes in patterning the land plant body plan following an ancient gene duplication. PLoS Genet..

[15] Truernit E., Haseloff J. (2007). A role for *KNAT* class II genes in root development. Plant Signal. Behav..

[16] Zhong R., Lee C., Zhou J., Ye M. (2008). A battery of transcription factors involved in the regulation of secondary cell wall biosynthesis in *Arabidopsis*. Plant Cell.

[17] Jia P., Xing L., Zhang C., Zhang D., An N. (2021). MdKNOX19, a class II knotted-like transcription factor of apple, plays roles in ABA signalling /sensitivity by targeting ABI5 during organ development. Plant Sci..

[18] Byrne M.E., Simorowski J., Martienssen R.A. (2002). *Asymmetric leaves1* reveals knox gene redundancy in *Arabidopsis*. Development.

[19] Venglat S.P., Dumonceaux T., Rozwadowski K., Parnell L., Datla R. (2002). The homeobox gene *BREVIPEDICELLUS* is a key regulator of inflorescence architecture in *Arabidopsis*. Proc. Natl. Acad. Sci. USA.

[20] Ragni L., Belles-Boix E., Gunl M., Pautot V. (2008). Interaction of *KNAT6* and *KNAT2* with BREVIPEDICELLUS and PENNYWISE in *Arabidopsis* inflorescences. Plant Cell.

[21] Yu L., Patibanda V., Smith H. (2009). A novel role of BELL1-like homeobox genes, *PENNYWISE* and *POUND-FOOLISH*, in floral patterning. Planta.

[22] Jasinski S., Piazza P., Craft J., Hay A., Tsiantis M. (2005). KNOX action in *Arabidopsis* is mediated by coordinate regulation of cytokinin and gibberellin activities. Curr. Biol..

[23] Du J., Mansfield S.D., Groover A.T. (2010). The Populus homeobox gene *Arborknox2* regulates cell differentiation during secondary growth. Plant J..

[24] Cheng X., Li M., Abdullah M., Li G., Lin Y. (2019). In silico genome-wide analysis of the pear (*Pyrus bretschneideri*) KNOX family and the functional characterization of *PbKNOX1*, an *Arabidopsis Brevipedicellus* orthologue gene, involved in cell wall and lignin biosynthesis. Front. Genet..

[25] Vollbrecht E., Veit B., Sinha N., Hake S. (1991). The developmental gene *Knotted-1* is a member of a maize homeobox gene family. Nature.

[26] Vollbrecht E., Reiser L., Hake S. (2000). Shoot meristem size is dependent on inbred background and presence of the maize homeobox gene, knotted1. Development.

[27] Jia P., Zhang C., Xing L., Li Y., Shah K., Zuo X., Zhang D., An N., Han M., Ren X. (2020). Genome-wide identification of the *MdKNOX* gene family and characterization of its transcriptional regulation in *Malus domestica*. Front. Plant Sci..

[28] Jia P., Xing L., Zhang C., Chen H., An N. (2021). MdKNOX15, a class I knotted-like transcription factor of apple, controls flowering and plant height by regulating GA levels through promoting the MdGA2ox7 transcription. Environ. Exp. Bot..

[29] Hackbusch J., Richter K., Müller J., Salamini F., Uhrig J.F. (2005). A central role of Arabidopsis thaliana ovate family proteins in networking and subcellular localization of 3-aa loop extension homeodomain proteins. Proc. Natl. Acad. Sci. USA.

[30] Gonzalez D.H. (2016). Plant transcription factors evolutionary, structural and functional aspects. Introduction to Transcription Factor Structure and Function.

[31] Lucas W.J., Bouche-Pillon S., Jackson D., Nguyen L., Baker L., Ding B., Hake S. (1995). Selective trafficking of KNOTTED1 Homeodomain protein and its mRNA through plasmodesmata. Science.

[32] Bueno N., Alvarez J.M., Ordás R. (2020). Characterization of the *Knotted1-Like Homeobox* (*KNOX*) gene family in *Pinus pinaster* Ait. Plant Sci..

[33] Li E., Bhargava A., Qiang W., Friedmann M., Forneris N., Savidge R., Johnson L., Mansfield S., Ellis B., Douglas C. (2012). The Class II *KNOX* gene *KNAT7* negatively regulates secondary wall formation in *Arabidopsis* and is functionally conserved in Populus. New Phytol..

[34] Wang S., Yamaguchi M., Grienenberger E., Martone P.T., Samuels A.L., Mansfield S.D. (2020). The Class II *KNOX* genes *KNAT3* and *KNAT7* work cooperatively to influence deposition of secondary cell walls that provide mechanical support to *Arabidopsis* stems. Plant J..

[35] Huang L.M. (2012). ATH1 and KNAT2 proteins act together in regulation of plant inflorescence architecture. J. Exp. Bot..

[36] Tsuda K., Hake S. (2015). Diverse functions of KNOX transcription factors in the diploid body plan of plants. Curr. Opin. Plant Biol..

[37] Yanai O., Shani E., Dolezal K., Tarkowski P., Ori N. (2005). *Arabidopsis* KNOXI proteins activate cytokinin biosynthesis. Curr. Biol..

[38] Hay A., Kaur H., Phillips A., Hedden P., Tsiantis M. (2002). The gibberellin pathway mediates knotted1-type homeobox function in plants with different body plans. Curr. Biol..

[39] Ye S.G., Zai W.S., Xiong Z.L., Zhang H.L., Ma Y.R. (2017). Genome-wide identification of gene family in tomato and their evolutionary relationship in *Solanaceae*. J. Nucl. Agric. Sci..

[40] Jie G., Yang X., Zhao W., Lang T., Tore S. (2015). Evolution, diversification, and expression of KNOX proteins in plants. Front. Plant Sci..

[41] Livak K.J., Schmittgen T.D. (2001). Analysis of relative gene expression data using real-time quantitative PCR and the 2^−ΔΔCT^ method. Methods.

[42] Liu M., Wu Z., Jiang F. (2015). Selection and validation of garlic reference genes for quantitative real-time PCR normalization. Plant Cell Tissue Organ Cult..

